# Otoscopy and imaging features of spontaneous temporomandibular joint herniation into the external auditory canal

**DOI:** 10.1259/bjro.20200005

**Published:** 2020-05-21

**Authors:** Jun-Hua Liu, Wen-Hu Huang, Jiang Hong Xu, Yin Liu, Yan Sha

**Affiliations:** 1 Department of Radiology, Eye and ENT, Hospital of Fudan University, Shanghai 200031, China; 2 Department of Otolaryngology, Eye and ENT, Hospital of Fudan University, Shanghai 200031, China

## Abstract

**Objective:**

To explore the otoscopy, CT and MRI features of spontaneous temporomandibular joint(TMJ）herniation(STMJH) into the external auditory canal (EAC) through the persistent foramen of Huschke (PFH).

**Methods:**

15 cases diagnosed STMJH were collected. The otoscopy, CT data of 15 cases and MRI data of 6 cases were retrospectively reviewed.

**Results:**

Otoscopy revealed a mass located in the anterior wall of the bony EAC that moved forwards and backwards during mouth opening and closing, respectively. CT showed a soft mass with bony defect in the anterior wall of the EAC, with no enhancement; the bony defect margin was well defined in all cases. The bone adjacent to the PFH was pressed and partially wrapped around the soft mass, as if “holding a ball,” in seven cases. Pseudobone shell around the soft mass was observed in eight cases. Six cases included MRI scans, which showed TMJ soft tissue herniated into the EAC.

**Conclusion:**

STMJHs have unique otoscopic, CT and MRI features. The examination strategy recommended is dynamic otoscopy and conventional CT, MRI can be chosen when the herniation is complicated by infection or otitis externa or when the patient has TMJ dysfunction; conservative management and follow-up observations are the main treatment strategy recommended.

**Advances in knowledge:**

Mechanical stress of TMJ on the EAC is thought to cause herniation and the special CT features, the location and size of the PFH, especially the location, are the major risk factors for TMJ herniation in patients with FH.

## Introduction

Structures of the temporomandibular joint (TMJ) can spontaneously herniate into the external auditory canal (EAC) through a congenital bone defect of the anterior EAC without inflammation, cholesteatoma, trauma or otologic surgery; this bone defect is called the persistent foramen of Huschke (PFH). At birth, the tympanic bone is incompletely developed, and only a U- or C-shaped tympanic ring fuses with the squamous portion of the temporal bone, with two prominences, one located interiorly and the other located posteriorly, arising from the tympanic ring. These two prominences grow progressively towards each other and meet, fusing at approximately one year old, resulting in the appearance of a foramen medial to the point of fusion. It is generally accepted that the foramen was first reported by Emil Huschke in 1844 and so named the foramen of Huschke (FH), but it is also known as the foramen tympanicum.^1,2^ The foramen becomes smaller as the tympanic plate grows through a membranous ossification process and usually closes before the age of 5 years; however, it may persist in varying sizes and shapes through life and occluded by a fibrous membrane, *i.e.* the PFH. The PFH is considered an anatomical variation that transmits no neural or vascular structures; in fact, it is an osseous defect that is not a true foramen.^3^

The PFH is a zone of reduced mechanical resistance that can be associated with significant complications, such as STMJH, the spread of infection or tumours between the EAC and TMJ, and ear injury.^4,5^ STMJH is extremely rare; there were some case report in the literature, however, the features and diagnostic strategy were not well described,^6^ we retrospectively studied 15 cases to explore the otoscopy and radiological features.

## Methods and materials

Ethical committee approval for this study was obtained. Two authors used our image reporting system database to retrospectively collect the data of all cases of a mass in the EAC, a bone defect in the EAC, or destruction of the EAC reported between January 2010 and March 2019 at the Affiliated ENT Hospital of Fudan University. The criteria for inclusion were the existence of a soft mass with a bony defect of the anterior wall of the EAC, no previous history of surgery or trauma to the temporal bone or mandible, and no history of inflammatory disease involving the TMJ or the EAC, including cholesteatoma of the EAC. Finally, 15 patients were included; there were eight males and seven females, ranging in age from 29 to 75 years with an average age of 56.5 years. The cases included six right ears and nine left ears. The course of disease ranged from 10 months to 11 years, and the main symptoms included ear fullness (seven cases), earache (five cases), otorrhagia (six cases), and tinnitus (three cases). Three patients underwent resection surgery, and two patients underwent biopsy surgery.

All patients underwent high-resolution CT of the temporal bone. Different scanners (SOMATOM Sensation 16, Siemens Medical System, Forchheim, Germany; SOMATOM Definition Edge, Siemens Medical System, Forchheim, Germany) were used over the lengthy period covered by our study. The scanning parameters were as follows: tube voltage, 120 kV; tube current, 240 mAs; collimator width, 6*0.75 mm; scanning range, from the top of the petrous bone to the mastoid tip. The thickness was 0.75 mm, and the slice increment was 0.3 mm; bony algorithms and soft tissue algorithms were used for reconstruction.

The acquired images were uploaded to the workstation for multiplanar reconstruction (MPR). The bony EAC was displayed by the sagittal image; the axis reference line was rotated to be perpendicular to the anterior wall of the EAC to acquire an axial image of the bony EAC. The sagittal reference line was rotated to be perpendicular to the anterior wall of the EAC on the axial image to acquire the sagittal image of the bony EAC. The maximum axial (length) and sagittal length (width) of the PFH were measured on the corresponding axial and sagittal reconstructions. The distance between the medial margin of the PFH and the tympanic sulcus was measured on the axial reconstruction. The shape of the PFH was observed by volumetric reconstruction and maximum density projection.

Six cases had contrast-enhanced MRI data obtained using a 3.0 T MRI scanner (Version, Siemens Medical System, Erlangen, Germany) with the following parameters: thickness, 2 mm; slice gap, 0.2 mm; matrix, 512 × 512; axial SE *T*1WI (repetition time, TR, 952 ms; echo time, TE, 12 ms); axial and coronal fast spin echo (FSE) *T*2WI (TR, 5000 ms; TE, 82 ms). After injecting the gadopentetate dimeglumine (Gd-DTPA, Magnevist, Bayer Schering), axial, coronal and sagittal fat-suppressed *T*1WI scans of the EAC were obtained.

Two doctors in the imaging department, both with over 10 years of experience, observed all images independently, and all measurements were averaged between the two observers.

15 patients underwent dynamic otoscopy; 11 patients had dynamic otoscopy reports, and 4 patients had additional dynamic otoscopy data obtained during outpatient follow-up visits after being included in this study.

## Results

### Otoscopy

**Figure 1.**
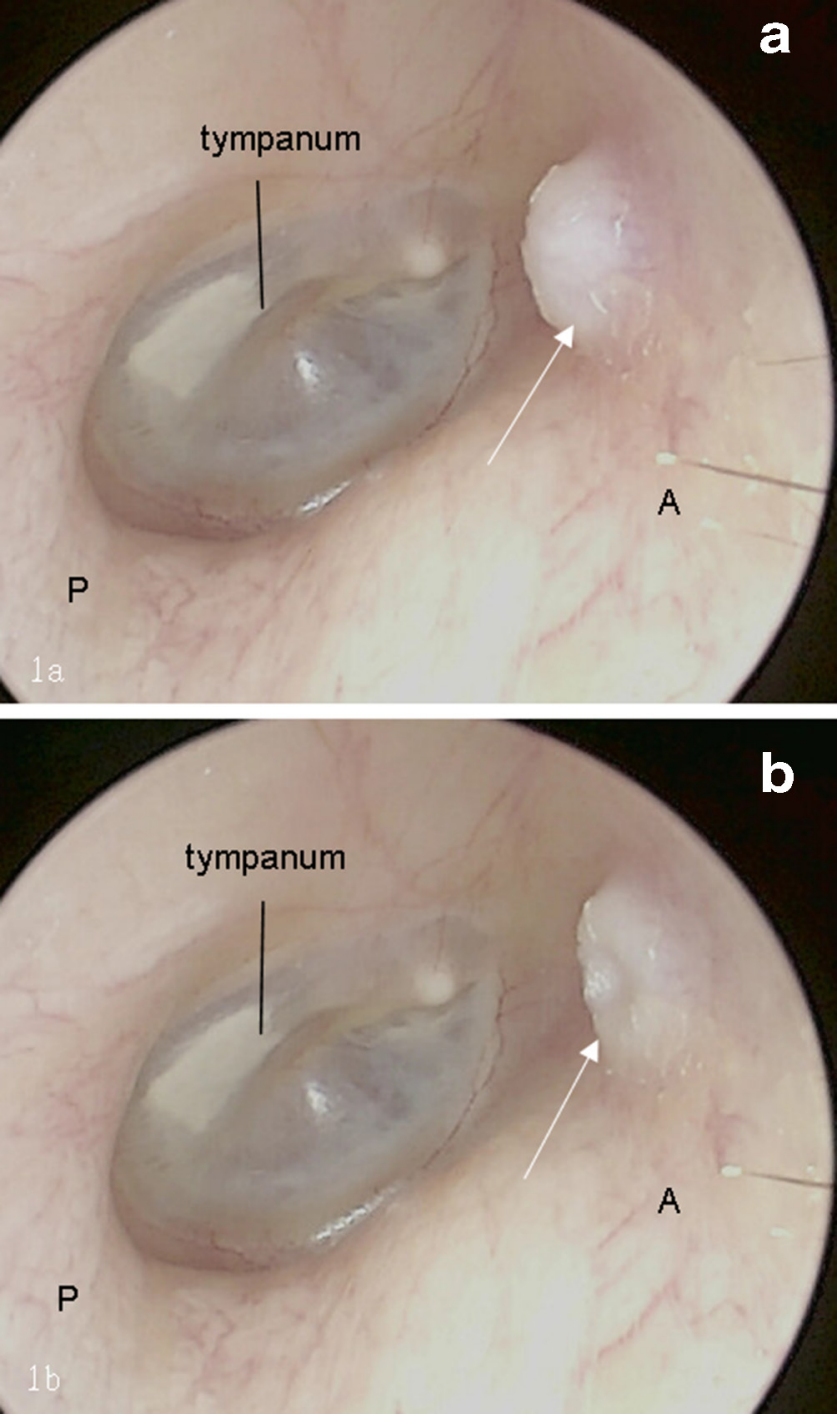
The appearance of the STMJH using dynamic otoscopy (white arrows). A: anterior wall; P: posterior wall. (a) In the closed-mouth position, the soft mass protruded into EAC. (b) In the open-mouth position, the mass moved forward and the skin of EAC invaginated, similar to a crater.

All cases had otoscopy data, which revealed a soft mass in the anterior wall of the bony EAC with a wide base and showed that the mass moved with mouth opening and closing. When opening the mouth, the mass moved forwards, producing an invagination of the skin of the EAC, similar to a crater, at the site of the soft mass. When closing the mouth, a dome-shaped mass moved backwards into the EAC (Figure 1). Some granulation tissue was observed on the surface of the mass or in the deep EAC in three cases. In two patients who had undergone resection surgery, during the outpatient follow-up period, slight movement of the skin of the EAC at the site of the previous herniation was observed during mouth closing and opening Figure 1.

In two patients who had undergone resection surgery, during the outpatient follow-up period, slight movement of the skin of the EAC at the site of the previous herniation was observed during mouth closing and opening Figure 1.

### CT and MRI

In all cases, a soft mass was observed adjacent to the bony defect in the anterior wall of the EAC. On the sagittal images in four cases, the sagittal diameter was larger and occupied most of the anterior wall; in seven cases, the sagittal diameter was located in the anterosuperior region of the wall; and in four cases, the sagittal diameter was located in the anteroinferior region of the wall. The mean length was 4.63 ± 2.24 mm (1.7–9.9 mm), the mean width was 3.91 ± 1.57 mm (1.5–7.5 mm), and the mean distance between the inner edge of the PFH and the tympanic sulcus was 7.35 ± 2.38 mm (3.5–11.7 mm).

**Figure 2.**
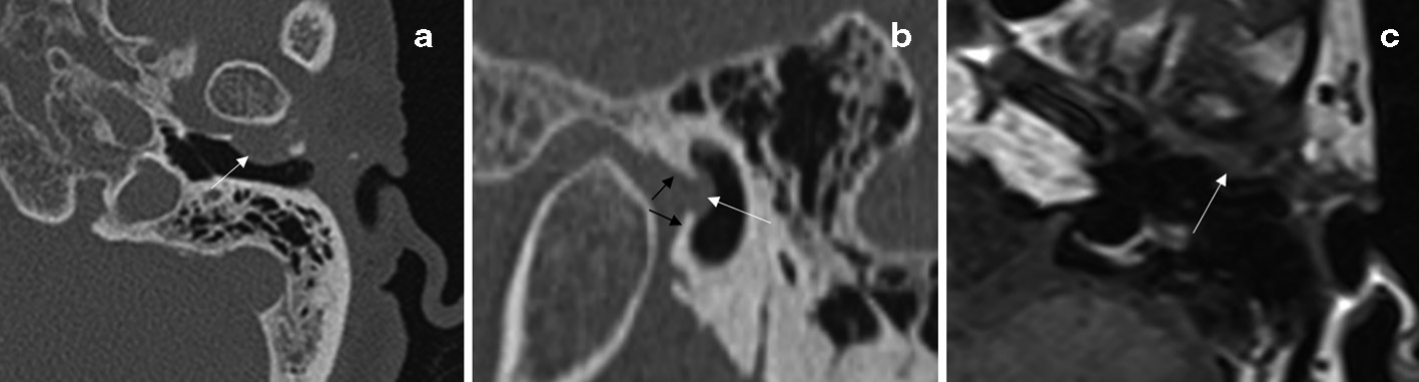
Images of STMJH (from the same patient). (a and b) The CT image, the image (a) showed a thin bony shell around the soft mass; the image (b) displayed the sign of “holding a ball” (black arrows). (c) The MR image, T1-weighted MR image in the axial plane, showed the bulging soft tissue protruded from the TMJ into the EAC

The shape of the PFH was round or round-like in eight cases and oval in seven cases on CT. The edge of the bony defect was smooth, there was no bony destruction or bony EAC enlargement in any case. In seven cases, the adjacent bone of the PFH protruded from the anterior to the posterior and partly encompassed the soft mass, as if it were holding a ball; in eight cases, there was a thin, incomplete bony shell around the soft mass. The soft mass had a broad base on the anterior wall of the EAC, which was a hemispherical, nodular or dome-shaped protrusion into the EAC, and there was some soft tissue in the EAC adjacent to the mass in three cases Figure 2

In six cases, MRI displayed a bony defect in the anterior wall of the EAC, and bulging soft tissue protruded from the TMJ into the EAC through the bony defect. The bulging soft tissue showed an isointense signal relative to the capsule of the TMJ on all sequences, and there was no enhancement after the intravenous administration of 0.2 ml/kg gadopentetate dimeglumine. In two cases, there was little fat tissue signal (Figure 2).

### Outcomes and follow-up data

Three patients underwent surgery for resection of the mass. In the surgery, the anterior wall of the EAC was exposed via a preauricular approach, the skin of the EAC was opened, and the continuity of the soft tissue mass with the white fibrous capsular tissue of the TMJ was observed protruding through the bony defect. One patient had a larger bone defect of approximately 6.1 × 5.5 mm in size that was located behind the head of the mandible. Herniation occurred again after the surgery, the patient underwent a post-operative CT and MRI scan, and the herniation was larger than the pre-operative hernia, but the bone defect as same as the pre-operative CT scan. Repair of the bone defect was suggested, however, the patient refused reoperation. The other two patients who underwent surgery did not experience recurrence over the next 5 years, follow-up CT images revealed a bony defect without the soft mass, and the bone defect as same as the pre-operative CT scan. Two patients underwent biopsy surgery, and histological analysis proved the sample to consist of normal fibrous connective tissue with inflammatory cells; these patients were followed as outpatients. The other 10 patients were followed as outpatients; the symptoms did not progress in any patient, and one patient achieved complete remission after 1 month. Two patients underwent CT again, which showed that the mass was not enlarging.

On histological analysis, the lesion proved to consist mostly of normal fibrous connective tissue with inflammatory cells and granulation tissue.

## Discussion

STMJH is extremely rare, and few case reports on STMJH have been published. Ertugrul^7^ and Park^8^ reported that the prevalence of STMJH was 0.4% in all samples and 2.9% or 27% in patients with PFH. The present study is the largest case series of STMJH.

PFH is mostly asymptomatic and may be overlooked on a CT scan or MRI. The overall pooled prevalence of PFH is estimated to be 14.9%.^5^ Typically, PFH is located in the anteroinferior region of the EAC, but occasionally appears in the anterosuperior region of the wall or even at the apex of the squamotympanic fissure or petrotympanic fissure,^4,9–14^ with 90.3%^5^ of cases occurring in the medial segment of the tympanic bone. The medial margin of the foramen is located very close to the margin of the tympanic sulcus with a mean distance of 1.0 mm^,3^ usually within 0.5 mm,^15^ immediately posterior and medial to the head of the mandible where the fat pat of the TMJ is located, and little of the stress of the TMJ transmits to this area. Although the PFH is present in many patients, only a minority experience a herniation.

This group included 13 patients with a STMJH located in the middle or outer segments of the bony EAC, and patients with a STMJH located in the anterosuperior wall. Although four cases were located in the anteroinferior wall, PFH near the head of mandible with herniation is more common. PFHs with herniation were larger than PFHs without herniation, as the pooled mean distance (PMD) of the total PFH length was 2.60 mm [95% confidence interval (CI): 2.43–2.77 mm], and the PMD of the total width was 2.40 mm (95% CI: 1.81–2.99 mm).^5^ A larger mean length (4.63 ± 2.24 mm) and mean width (3.91 ± 1.57 mm) of the PHF were observed in this group. The location and size of the PFH, particularly the location, are the major risk factors for TMJ herniation in patients with PFH.

Mechanical stress of the TMJ is thought to cause herniation.^14,15^ As the pressure of TMJ movements are transmitted to the weak anterior wall of the EAC with a PFH, the foramen enlarges over a long period and the joint capsule and retrodiscal tissue or disc are pulled through the foramen, prolapsing or spontaneously herniating into the EAC, which causes the unique CT signs. If the bony wall around the PFH is thinner, the soft tissue around the hernia is also compressed backwards, causing the adjacent wall to press backwards and the formation of the edge of the herniation in the form of a “ball being held” on CT scans. Focal weakness of the former FH and even the presence of a sieve-like osseous plate^16^ during the formation of the hernia leads to backwards displacement of the TMJ soft tissue, imposing backwards pressure on the weak bony wall and causing a fracture and the subsequent formation of an incomplete pseudobony shell around the surface of the herniated tissue, as shown in CT images.

The symptoms of STMJH are non-specific; however, the otoscopy findings are characteristics. Dynamic otoscopy of STMJH perfectly corresponds with the definition of the herniation. Therefore, although the diagnosis of the herniation can be confirmed using a simple clinical examination or dynamic otoscopy, contrast-enhanced MRI can also diagnose the STMJH, but is usually unnecessary. MRI is suggested when the herniation is complicated with an infection, otitis externa, or TMJ dysfunction. CT is very sensitive in detecting the osseous defect, the size and location of the herniation, and information about the bony EAC to help doctor determine the treatment. However, contrast-enhanced CT is not necessary. If a soft mass with a focal bone defect in the anterior wall of the EAC is observed on CT, dynamic otoscopy is required, and thus the strategy of the examination was dynamic otoscopy and conventional CT.

STMJH causes stenosis of the EAC, interfering with the discharge of secretions and water. The skin of the EAC becomes more easily damaged, and herniation is often accompanied by inflammatory lesions in the EAC, such as eczema and granulation tissue^17^; thus, the differential diagnosis of inflammatory lesions in the EAC is difficult. If extensive inflammation of the EAC and herniation is observed, dynamic otoscopy is often ignored, and the condition is often misdiagnosed as otitis externa. A CT scan can show the herniation and bony defect and is useful for the differential diagnosis.

The tympanic plate has a relatively poor vascular supply, and defects in the anterior bony wall of the EAC may be caused by various pathological processes, such as cholesteatoma, necrotizing external otitis, temporal bone trauma, rheumatoid arthritis of the TMJ, otologic surgery, and radiation treatment for head and neck cancer, particularly nasopharyngeal cancer. Destruction of the anterior EAC wall may cause secondary herniation.^18^ However, the bony destruction observed in a CT image is different from the appearance of FH; the destroyed bony edge in a cholesteatoma is often compressed forwards and does not have a bony shell around the soft tissue. Inflammatory disease and radiation-induced osteonecrosis often lead to erosive osteoclasia, in which the shape of the destroyed bone is irregular and the edge is not clear; dead bone may even be present. In these cases, most of the anterior wall of the EAC moves backwards or forwards during the dynamic otoscopy examination and a certain soft mass is not observed.

Benign tumours of the bony EAC have a similar appearance to herniations under a static otoscope, while the intact bone wall may appear different on a CT scan. In the case of a benign tumour that is located at the PFH in the EAC, conventional CT is unable to be used for the differential diagnosis of herniation; however, dynamic otoscopy and MRI easily facilitate the diagnosis, as the mass will not move when the patient opens or closes his/her mouth.

We recommend conservative management and follow-up observations of the herniation as the main treatment strategy. A substantial period of rest and abstinence should be advised. A biopsy is unnecessary, as a definitive diagnosis does not require a biopsy. If a patient has an excessively large hernia, severe ear fullness or repeated infection of the ear canal, the patient could be treated with surgery, which depends on the patient’s willingness and suitability for surgery. If the symptoms include TMJ pain, malocclusion, or tinnitus while chewing, a consultation with oral and maxillofacial surgeons is necessary.^19^ Surgical treatment includes hernia resection and bone defect repair. Various materials have been used to close this type of defect, such as fascia,^20^ cartilage,^6,21–23^ autologous bone chips,^11^ polypropylene plate, collagen mesh,^24^ and titanium mesh.^25^

## Conclusions

Spontaneous herniation of the TMJ into the EAC is more commonly located in the middle or outer segments of the bony EAC. The dynamic otoscopy, CT and MRI features are unique, and it can be confirmed by dynamic otoscopy or contrast-enhanced MRI. MRI scan is usually unnecessary, and thus the strategy of the examination is dynamic otoscopy and conventional CT. Conservative management and follow-up observations are recommended as the main treatment strategy.
